# The Sex Difference in the Pathophysiology of Preterm Birth

**DOI:** 10.3390/cells14141084

**Published:** 2025-07-16

**Authors:** Gain Lee, Gisela Martinez Andrade, Young Ju Kim, Dilly O. C. Anumba

**Affiliations:** 1 Graduate Program in System Health Science and Engineering, Ewha Womans University, Seoul 03760, Republic of Korea; lgi012@ewhain.net; 2 Division of Clinical Medicine, School of Medicine & Population Health, Faculty of Health, The University of Sheffield, Jessop Wing, Tree Root Walk, Sheffield S10 2SF, UK; gisela.martinezandrade@sheffield.ac.uk

**Keywords:** preterm birth, placental dysfunction, sex-specific manner, inflammation, oxidative stress

## Abstract

Preterm birth (PTB) refers to a labor before 37 gestational weeks. This is a major global contributor to neonatal morbidity and mortality. Although fetal sex is frequently treated as a confounding variable in PTB research, relatively few studies have conducted sex-stratified analyses to investigate how male and female fetuses may respond differently to various intrauterine exposures. This represents an underexplored area with important implications for understanding fetal sexual dimorphism-specific vulnerability to adverse pregnancy outcomes. Understanding the role of fetal sex differences in the pathophysiology of preterm birth (PTB) regarding processes such as inflammation, placental dysfunction, and oxidative stress is crucial. These delicate processes are tightly interrelated, but also independently contribute to pregnancy complications. Recognizing fetal sex as a biological variable for such processes is essential for improving mechanistic insight, providing refined predictive models.

## 1. Introduction

Preterm birth (PTB), defined by the World Health Organization as a delivery before 37 weeks of gestation [1], affects 9.9% of births worldwide and remains a leading cause of neonatal mortality and morbidity [2]. The insufficient development of the placenta contributes to PTB, and resulting newborns are at risk of short-term complications such as long-term physical and neurodevelopmental disorders, which are noted to affect male infants disproportionately [3].

Approximately two-thirds of PTBs are spontaneous PTBs (sPTB), while the remaining 30–35% of PTBs are medically indicated, sometimes associated with conditions such as preterm premature rupture of the membrane (pPROM), intrauterine infections, or inflammation-driven preterm labor [4,5,6]. These proportions may vary based on population characteristics and geographic regions. Most studies report high negative predictive values of these tests, but their positive predictive values remain low and inconsistent.

Emerging evidence suggests that PTB results from a complex interplay of multiple pathologic mechanisms, including ascending infection triggered by systemic inflammation, vaginal dysbiosis, endocrine dysregulation, and oxidative stress [7,8,9,10,11]. These pathologies are further influenced by environmental factors (e.g., particulate matter, microplastics, heavy metals, etc.), maternal behaviors (e.g., smoking, alcohol consumption, and high-fat diet), stress, socioeconomic status, and a previous PTB history [12,13,14].

Recent studies also suggest that maternal endocrine fluctuations—such as altered levels of progesterone (P4), estrogen, oxytocin, and fetal sex hormones—may contribute to sPTBs [4,15]. However, fetal sex as a biological confounding variable is still underrecognized in many observational studies, despite the accumulating evidence that male fetuses are more susceptible to adverse intrauterine conditions [16].

**Figure 1:**
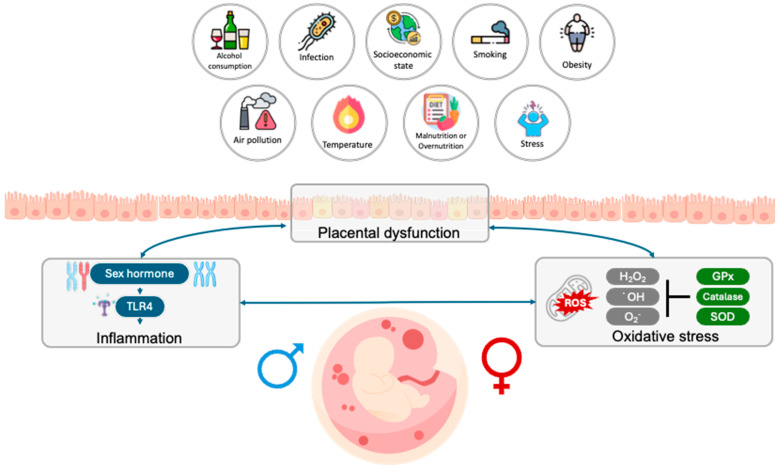
External factors affecting pathogenic preterm birth. The following scheme illustrates how related risk factors affect the pathophysiology of PTB. Several factors have been identified as contributing to placental dysfunction, including external environmental factors (e.g., particulate matter, heavy metals, and temperature), as well as infections (e.g., bacteria), smoking, malnutrition, a high-fat diet, stress, socioeconomic status, and ethnicity. These factors have been linked to being associated with increased inflammation, oxidative stress, and other related complications. It is evident that hormonal signaling, specifically estrogen, testosterone, and progesterone (P4), plays a pivotal role in the initiation of labor. Spontaneous preterm labor leading to preterm birth is a complex syndrome consisting of several diseases, each of which can be an independent initiating factor in the path to labor induction. All of these disease processes can induce inflammation and oxidative stress. The figure was created with BioRender.com, accessed on 2 June 2025.

In this review, we aim to synthesize the current knowledge on the sex-specific mechanisms underlying PTBs, with a particular focus on how inflammation, placental dysfunction, and oxidative stress differentially affect pregnancy outcomes based on fetal sex (Figure 1).

## 2. Environmental Factors Influence Preterm Birth Prevalence According to Fetal Sex

The “Developmental Origins of Health and Disease (DOHaD)” hypothesis posits that prenatal exposure to environmental and physiological stressors can program long-term health outcomes [17]. Prenatal exposures, including environmental pollutants and endocrine disruptors, have been shown to influence not only fetal development but also the sex ratio at birth [18,19]. A large-scale cohort study from the Netherlands involving 1,736,615 singleton pregnancies found that a male fetus is a relevant risk factor for sPTB with intact membranes [20]. Additionally, male neonates in low-risk populations exhibit lower umbilical cord blood pH and lower APGAR scores at both 1 and 5 min than their female counterparts, indicating greater vulnerability to perinatal stress [21]. This phenomenon presents a similar tendency to neonatal adverse outcomes. Low birth weight [22], respiratory distress syndrome (RDS) [23], bronchopulmonary dysplasia (BPD) [24], and periventricular–intraventricular hemorrhage (PIVH) [25] showed higher prevalence in male infants.

Sex-based disparities in pregnancy outcomes have been consistently observed across various physiological domains (Table 1). Sex- and age-related brain differences showed that males have larger overall volumes and more white matter. In contrast, females exhibit more cortical gray matter and region-specific increases, particularly in the left anterior cingulate and superior temporal gyrus [26]. At the molecular level, prenatal exposure to air pollution is associated with the heightened activation of immune pathways in females, while males demonstrate an increased expression of genes related to synaptic signaling and mitochondrial function during the last month of gestation [27]. These findings suggest distinct biological strategies in male and female fetuses when adapting to intrauterine stress.

Despite the growing evidence, the impact of fetal sex on pregnancy complications and environmental interactions remains underexplored in many studies. The greater consideration of sex as a biological variable is essential for advancing our understanding of PTB pathogenesis, and for developing sex-specific preventive strategies.

## 3. Sex-Specific Modulation of Inflammation in Preterm Birth

### 3.1. Inflammatory Mechanism in Pregnancy

Among several physiological processes involved in pregnancy, inflammation plays a paradoxical role. While excessive or dysregulated inflammation is the primary trigger for the onset of PTB [35], controlled inflammation responses are essential for implantation and the successful maintenance of pregnancy, as well as labor in humans, mice, and opossums [36].

The cytokines, chemokines, and prostaglandins present in the seminal fluid initiate the recruitment of leukocytes and stimulate pro-inflammatory cytokines within the uterus [37,38]. During normal pregnancy, the maternal immune tolerance mechanism suppresses fetal rejection by recognizing paternal antigens expressed in fetal tissues. Maternal immune tolerance has been shown to mitigate the risk of fetal rejection and supports the remodeling of spiral arteries (SpAs) and placentation [35]. While physiological inflammation supports implantation and placentation, an imbalanced or dysregulated pathway can shift immune responses toward pathological outcomes, such as PTB.

### 3.2. Inflammatory Dysregulation and Preterm Birth

The onset of parturition is associated with a physiologic inflammatory cascade [39]. One of the critical components in this process is the activation of toll-like receptors (TLRs), which detect pathogen-associated molecular patterns (PAMPs) and damage-associated molecular patterns (DAMPs) originating from microbes and necrotic cells, respectively [40,41]. This TLR activation initiates inflammasome formation, which stimulates the increased expression of pro-inflammatory cytokines, chemokines (e.g., tumor necrosis factor (TNF)-α, interleukin (IL)-1β, IL-6, IL-8, and granulocyte–macrophage colony-stimulating factor (GM-CSF)) and leukocytes (macrophages, monocytes, and neutrophils). Concurrently, anti-inflammatory cytokines (IL-10, TGF-β, etc.) are decreased in the placenta and amniotic cavity [42,43,44,45].

The microbial invasion of the amniotic cavity (MIAC) or intra-amniotic infection, often following ascending infection from the lower genital tract, can lead to sterile inflammation and pPROM, and ultimately PTB [46,47,48]. A recent study demonstrated that TLR4 signaling in terms of endothelial cells can induce IL-6 secretion in perivascular stromal cells via nuclear factor kappa-light-chain-enhancer of activated B cell (NF-κb) activation, which also involves the IL-10/STAT3 axis [49,50]. NF-κb activation induces genes associated with uterine contractility and cervical ripening, such as the prostaglandin (PG) F2α receptor, connexin-43, the oxytocin receptor, and cyclooxygenase 2 (COX-2). The accumulation of pro-inflammatory cytokines promotes PG synthesis and membrane rupture—key events in the pathophysiology of pPROM and PTB [51,52].

### 3.3. Fetal Sex-Based Modulation of Inflammatory Responses

#### 3.3.1. Sex Differences in Immune Regulation

**Figure 2:**
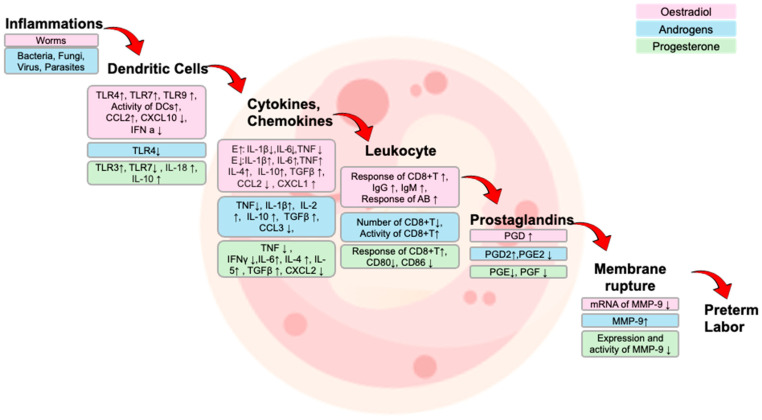
Modulation of inflammation-related pathway by sex steroid hormones in response to infection. This schematic illustrates the immune signaling cascade initiated by pathogen recognition and its modulation by sex steroid hormones―oestradiol (pink), androgens (blue), and progesterone (green). PAMPs (e.g., from bacteria, viruses, fungi, and parasites) are recognized by dendritic cells, leading to the activation of toll-like receptors (TLRs) and the downstream adaptor myeloid differentiation primary response 88 (MyD88). This signaling induces the expression of pro-inflammatory cytokines (e.g., interleukin (IL)-1β, IL-6, tumor necrosis factor-α (TNF-α), interferon-γ (IFN-γ)) and chemokines (e.g., C-C motif chemokine ligand (CCL2), CCL3, C-X-C motif chemokine ligand 8 (CXCL8)), which promote leukocyte recruitment. Leukocytes contribute to the production of prostaglandins (e.g., PGE_2_, PGF2α, PGD2) via cyclooxygenase-2 (COX-2), which in turn activates matrix metalloproteinase-9 (MMP-9). This pathway may facilitate extracellular matrix remodeling and placental membrane contraction, events implicated in the initiation of labor and preterm birth. Sex steroid hormones differentially regulate these processes: oestradiol upregulates pro-inflammatory mediators and prostaglandin synthesis; androgens and progesterone generally suppress TLR signaling, cytokine/chemokine expression, COX-2 activity, and MMP-9 activation. Abbreviations: AB, antibody; CCL, C-C motif chemokine ligand; CXCL, C-X-C motif chemokine ligand; DCs, dendritic cells; E, oestradial; IFN-γ, interferon-γ; Ig, immunoglobulin; IL, interleukin; MMP-9, matrix metalloproteinase-9; PGD, prostaglandin; PGE, prostaglandin E; PGF, prostaglandin F; TGFβ, transforming growth factor β; TLR, toll-like receptor; TNF, tumor necrosis factor.

Not only does maternal inflammation signaling contribute, but the fetus itself plays a role in initiating the signal of labor. In particular, immune responses in utero are influenced by both the sex chromosome complements (XX vs. XY) and sex steroid hormones (estrogen, progesterone, and androgen) [53]. According to a comprehensive review by Klein et al., males and females exhibit distinct immunological profiles across the life stages, including fetal development [54]. These differences affect the magnitude and type of both innate and adaptive immune responses and can influence the risk of PTB (Figure 2).

#### 3.3.2. Sex Chromosomal Contribution to Immune Expression

The Y chromosome contains the sex-determining region Y (SRY) gene, which drives testis development and testosterone synthesis. In its absence, XY– (SRY- deficient) mice develop as phenotypic females with ovaries despite having a male chromosomal complement [55]. This difference may be related to decreased Th2 cytokines (IL-4, IL-5, IL-13), and increased levels of IL-13Rα2 on macrophages (CD11b+) and dendritic cells (CD11c+) [55]. In a mouse model using SRY-deficient males, CD4+ T cells showed a significantly increased expression of the histone demethylase KDM6A, which is implicated in autoimmune disease [56,57]. Furthermore, as men age, the Y chromosome may diminish from their immune cells, which increases their risk of death from cancer [58]. The lower TLR7 expression in male cells has been associated with increased susceptibility to infections such as SARS-CoV-2 and neonatal respiratory syncytial virus [59,60]. Abdel-Hafiz et al. revealed that male patients with a Y loss mutation promote the severity and number of other mutations, increasing the dysfunction of CD8+ T cells [61].

Conversely, in the case of females, a pair of X chromosomes is inherited—one maternal and one paternal—resulting in a diverse set of gene expressions [62]. To compensate for the imbalance due to double X chromosomes, one X chromosome undergoes random inactivation during early embryogenesis, a process known as X chromosome inactivation (XCI) [63]. This results in a cellular mosaicism state, allowing a balanced gene expression irrespective of sex [64]. Escape from XCI in certain loci contributes to allelic diversity, particularly in immune genes, and influences inflammatory responses [65]. Single-cell RNA sequencing identified 24 and 49 candidate escapees from fibroblasts and lymphoblasts, respectively [66]. The TLR7, a crucial component in antiviral defense, escapes XCI and is expressed at higher levels in female cells [67]. Taken together, these sexually dimorphic immune responses contribute to altered immune expression and disease susceptibility through sex chromosome-linked genetic and epigenetic differences.

#### 3.3.3. PTB-Related Immune Modulation by Fetal Sex Difference Manner

Evidence has suggested that both type I and II interferon signaling, as well as humoral responses, are more robust in females than in males across several species [53]. For instance, cord blood immunoglobulin (Ig) E levels—a marker for allergic sensitization—are typically higher in male neonates than in females [68]. In a clinical study, men who were treated with gonadotropin-releasing hormone (GnRH) antagonist experienced a decrease in T_reg_ (CD4+CD25+) without an alteration in the CD4+:CD8+ ratio. Additionally, medical castration increased natural killer (NK) cell activity while reducing IFN-γ production in CD8+ T cells [69], indicating that the hormonal milieu significantly shapes immune cell profiles. Female trophoblasts show a sensitivity in response to various compounds, displaying upregulated chemokines, such as CCL3, CCL4, and CXCL8 [70]. The migration of NK cells and monocytes is regulated by the binding of CCL3 and CCL4 to decidua-expressed receptors CCR1 and CCR5, respectively. Sun et al. suggested that the Th2 response is induced by increased M2, provoked by raised CCL13 and RGS1 in female Hofbauer cells. CCRL2, LGALS13, and LGALS14, which are regarded as regulating mitochondria, immune, and pregnancy maintenance-related transcripts, were highly upregulated on chromosome 19 in male chorionic villus between 11 and 16 weeks of gestation [71]. In male trophoblast cells, HLA-C, MUC15, NOTUM, SNHG19, and SNHG25 were upregulated [70]. The higher vulnerability of male neonates to infections, such as neonatal sepsis and meningitis, further highlights inherent sex-based differences in immune competence [72].

Furthermore, 16s rRNA sequencing studies have suggested that the composition of the vaginal microbiome is associated with preterm or term birth [73,74]. A high risk of PTB might be associated with a lack of *Lactobacillus* spp. in the cervicovaginal fluid [74,75]. N-glycosylation, which is a major immune-modulatory feature of the cervicovaginal fluid component, is influenced by pregnancy and immune state [76]. Estrogens, which increase during pregnancy, can modulate IgG glycosylation [77]. Since glycosylation patterns affect microbial adhesion to epithelial cells [78], the estrogen-mediated accumulation of glycogen in the vaginal epithelium may indirectly regulate microbial colonization and immune responses. Together, sex-specific immune modulation—mediated by interferon signaling, hormonal regulation, and trophoblast gene expression—contributes to the differential susceptibility to PTB. Additionally, cervicovaginal microbiome composition and glycosylation profiles may further reflect fetal sex-dependent differences.

## 4. Placental Dysfunction in Preterm Brith

### 4.1. Placental Formation and Function in Pregnancy

The placenta is a critical organ that forms 5–6 days after fertilization and serves as the interface between the maternal and fetal environments throughout gestation [79]. After implantation, trophoblast cells differentiate into two distinct lineages: villous and extravillous [80]. Villous cytotrophoblasts fuse to form multinucleated syncytiotrophoblasts, which comprise the outer epithelial layer of the chorionic villi and play a key role in nutrient change and hormone secretion [81]. The SpAs undergo extensive remodeling from early pregnancy through to approximately 22 weeks of gestation to facilitate proper placental perfusion [82].

The placenta is responsible for the exchange of oxygen, nutrients, hormones, and waste along with cells, and various signaling molecules, including nucleic acid, extracellular vesicles (EVs), and exosomes [80,83]. The syncytiotrophoblasts also produce steroid hormones, glycoproteins, and cytokines, all of which are essential for fetal development and immune modulation [84]. For instance, 17β-estradiol and P4 regulate changes in the endometrium and influence the expression of adhesion molecules, growth factors, and human chorionic gonadotropin (hCG), thereby facilitating successful implantation [85]. hCG, in turn, stimulates the ovarian secretion of P4, which maintains the secretory activity of the endometrium and downregulates progesterone receptor A (PRA) expression on maternal epithelial cells [79,84].

### 4.2. Placenta Dysfunction and Role in Preterm Brith

Placental dysfunction—also referred to as placental insufficiency—occurs when the placenta fails to adequately provide oxygen and nutrients to a developing fetus [86]. The dysfunction is recognized as a key contributor to adverse pregnancy outcomes, including PTB, miscarriage, stillbirth, and placental abruption [3,86,87,88]. In particular, impaired placental development or the insufficient remodeling of SpAs leads to increased vascular resistance and decreased placental blood flow [89], which may trigger disorders such as preeclampsia (PE), a hypertensive condition associated with proteinuria during pregnancy [90].

Placental dysfunction is affected by not only inflammation but also oxidative stress. Interestingly, placental dysfunction also induces inflammation and/or oxidative stress. In normal gestation, placental senescence is a progressive process, with syncytiotrophoblasts showing increasing markers of oxidative stress and cellular aging [91]. Placental tissues from cesarean sections showed elevated inflammation markers (IL-1α/β, IL-6, IL-8, CCL2, and TNF-α) when they were exposed to a state of hypoxia [92]. Oxidative stress, along with the activation of pathways such as G-protein-coupled estrogen receptor 1 (GPER1), has been implicated in advancing placental senescence [91,93]. Furthermore, placental dysfunction is closely associated with pPROM and placental abruption, a major cause of sPTB [88,93,94,95].

### 4.3. Fetal Sex Hormone Contributing to Placental Dysfunction

#### 4.3.1. Sex Difference Effects on Placental Structure

Sex-specific genetic and epigenetic mechanisms significantly influence placental structural development. The differential expression of genes on the X and Y chromosomes contributes to variations in placental architecture and cellular composition. For instance, male placentas exhibit a relative downregulation of the ITGβ8 gene, which promotes angiogenesis and tissue invasion, both of which are vital during early gestation [96]. This downregulation may impair placental vascularization and nutrient exchange capacity, potentially predisposing male fetuses to suboptimal intrauterine environments.

Further transcriptomic profiling has revealed that the expression of genes related to immune regulation, such as those involved in graft-versus-host disease and inflammatory responses, is elevated in male placental villi [97,98]. This higher expression of genes related to immune tolerance and pregnancy maintenance in female placentas further suggests that male fetuses may be more vulnerable to early pregnancy loss due to impaired placentation [99].

The development of sequence technologies has reinforced more detailed transcriptional and epigenetic analyses of placental proportion. Sadiqi et al. used 450K DNA methylation to investigate whether the timing of PM_2.5_ exposure during pregnancy differentially affects the overall placental cell composition by fetal sex [100]. In male infants, first-trimester (T1) PM_2.5_ exposure was associated with a decreased proportion of syncytiotrophoblasts and increased trophoblasts. In females, second- (T2) and third- (T3) trimester exposure led to a decreased proportion of nucleated red blood cells (nRBCs) [100]. Rodent studies provide further evidence to support this assertion. At embryonic day (E) 15, the placental labyrinth zone fetal and maternal blood space had reduced volume in all compartments compared with gestational age-matched males [101]. The reduced E15 female labyrinth volume was associated with an overall decrease in the expression of differentiation- and growth-related genes, including MEST, GCM1, SYNA, insulin-like growth factor (IGF)2, and IGF2r [101].

Comprehensively, sex-specific genetic, epigenetic, and hormonal influences affect structural and cellular differences in the developing placenta, and male fetuses are more susceptible to placental dysfunction and intrauterine disease due to impaired angiogenesis and immune regulation.

#### 4.3.2. Growth Strategy According to Fetal Sex

Sexual dimorphism in fetal adaptive strategies has been extensively documented, with compelling evidence indicating that male and female fetuses exhibit divergent growth responses to intrauterine stressors. Male fetuses tend to adopt a “growth-priority” mode, maintaining somatic development even under suboptimal placental function. In contrast, female fetuses more often implement a “placental adaptation” strategy, showing growth in favor of sustaining viability in adverse conditions [102,103].

**Figure 3:**
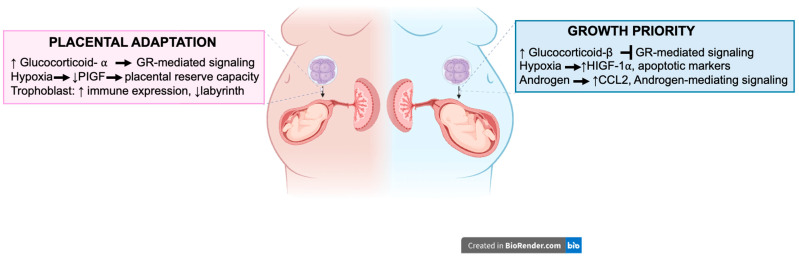
Sex-specific placental adaptation and fetal growth strategies in response to intrauterine stress. The schematic illustrates differential placental and fetal responses in pregnancies carrying male (blue) versus female (pink) fetuses. Female placentas exhibit adaptive mechanisms including increased glucocorticoid receptor (GR)-mediated signaling, reduced placental growth factor (PlGF) expression under hypoxia, and enhanced trophoblast immune modulation with reduced labyrinth development. Male fetuses exhibit a growth-prioritizing strategy characterized by decreased GR-mediated signaling, increased expression of hypoxia-inducible factor (HIF)-1α and apoptotic marker, and androgen-driven signaling. Namely, rather than diminishing the placental adaptability of male placentas under adverse intrauterine conditions, an optimal environment for fetal growth is facilitated. The figure was created with BioRender.com, accessed on 2 June 2025.

Sex chromosome complement (XX vs. XY) significantly contributes to placental development and the concentration of circulating sex hormones [53]. The distinction is partly rooted in the early expression of the SRY gene, which initiates testis development and testosterone production before significant estrogenic activity arises [104,105]. These sex-specific hormonal profiles may contribute to divergent placental responses, with male placentas displaying reduced resilience under conditions of maternal stress (Figure 3).

Epidemiological studies support this; female fetuses are more commonly associated with preterm PE (delivered < 37 weeks of gestation) and very preterm PE (delivered < 34 weeks of gestation) [106]. However, term PE (delivered > 37 week of gestation) is more often linked to male fetuses [16]. These differences may mean that pregnancies susceptible to PE due to poor placental development are more prone to result in miscarriage if the fetus is male [106]. Consequently, surviving male fetuses may potentially represent a healthier subset, explaining the female predominance in preterm PE and male predominance in term PE. Interestingly, maternal post-reproductive lifespan may also be affected by fetal sex. Several studies have suggested that bearing male offspring may reduce maternal longevity, possibly due to greater physiological demands during pregnancy [107,108].

The placental abruption accounts for approximately 5% of all PTBs [109], and a male fetus has been identified as a significant risk factor (OR 1.38; 95% CI 1.12–1.70) [110]. Furthermore, spontaneous abortion risk is approximately 30% higher for a male fetus, resulting in male-to-female ratio of 1.32 in chromosomally normal cases [111]. Research indicates that the anatomical sex ratio in spontaneous abortion is 1.25, indicating a male fetus excess that is apparent at all gestational ages and all sizes [112]. These findings reinforce the notion that the male fetus is more susceptible to placental dysfunction. This aligns with observations linking male fetal sex to other placental-related complications, including PTB.

#### 4.3.3. Transcriptional and Epigenetic Differences

Recent advancements in high-throughput sequencing technologies have enabled the analysis of sexually dimorphic gene expression patterns in placental tissues. Transcriptomic analyses have shown that female placentas exhibit a higher expression of genes associated with immune tolerance and regulators of maternal–fetal immune interaction [99]. In contrast, women carrying male fetuses showed increased rates of chronic inflammatory placental lesions [113]. Male placentas exhibit heightened transcriptional activity in pro-inflammatory and immune rejection pathways, potentially contributing to poorer placental outcomes [97]. Ferrous iron (Fe^2+^) is essential for the KDM3A-mediated histone demethylation necessary for SRY during male gonadal development [114]. The disruption of iron metabolism, via Tfrc deletion or maternal iron deficiency combined with a KDM3A variant, impairs SRY expression and causes male-to-female sex reversal in mice [114]. These experiments demonstrate epigenetic programming, which can change and potentially influence placental function and the fetus’s ability to adapt to inflammatory stressors.

Moreover, hormonal signaling exhibits sexually dimorphic transcriptional modulation. Androgens are critical for the development of the male reproductive tract between gestational weeks 7 and 12 and also serve as precursors for estrogen biosynthesis in both sexes [115,116]. The androgen receptor (AR)-associated gene ARMCX3, which is upregulated in early male placental development, plays a role in mitochondrial dynamics and trophoblast function, with implications for long-term placental performance [97,117].

Additionally, the expression of pregnancy-associated plasma protein (PAPP)-A and free β-hCG, primarily secreted by syncytiotrophoblasts, is significantly higher in female pregnancies [118,119,120]. Yaron et al. reported that maternal serum levels of both PAPP-A and β-hCG were significantly increased in female fetuses compared with male fetuses [118]. This heightened expression in females may enhance endocrine support, immune tolerance, and placental adaptation by modulating trophoblast invasion and maternal vascular remodeling.

Comprehensively, these data suggest that fetal sex influences not only the structural configuration of the placenta but also its transcriptomic and hormonal milieu, thereby shaping differential susceptibilities to gestational complications such as PTB, PE, and spontaneous abortion. Male fetuses, due to a more rigid and growth-focused transcriptional program, may exhibit less plasticity in response to environmental insults, rendering them more vulnerable to placental dysfunction.

## 5. Oxidative Stress as a Trigger for Preterm Birth

### 5.1. Balanced Oxidative Stress in Normal Pregnancy

The reactive oxygen species (ROS), which include superoxide radicals (O_2_^−^), hydrogen peroxide (H_2_O_2_), and hydroxyl radicals (HO˙), are the byproducts of aerobic cellular metabolism and serve as important signaling molecules in various physiological processes such as cell proliferation, autophagy, and inflammation modulation [121,122]. Maintaining a balanced level of ROS is crucial for normal pregnancy progression, including placental development and the timely initiation of labor.

As pregnancy progresses, there is an increase in mitochondrial activity in response to elevated oxygen requirements, particularly between weeks 10 and 12 of gestation. At this time, chorionic villi detach from the maternal SpAs, allowing maternal blood to flood the intervillous space and precipitating a substantial rise in O_2_ tension [123,124]. Notably, high levels of H_2_O_2_ are synthesized at the perivitelline space of each zygote, suggesting that H_2_O_2_ plays a vital role in forming a protective layer around the fertilized egg [121,125]. In response to ROS accumulation and to prevent oxidative damage, the placenta upregulates the antioxidant defense system. It is evidential that major placental antioxidants include heme oxygenase (HO)-1 and -2, superoxide dismutase (SOD), catalase, and GPx [126]. Collectively, these exert their antioxidant effects by neutralizing ROS, thus ensuring the maintenance of redox homeostasis, a state that is essential for the proper functioning of the placenta and the development of the fetus.

### 5.2. Imbalanced Oxidative Stress and Adverse Pregnancy Outcomes

**Figure 4:**
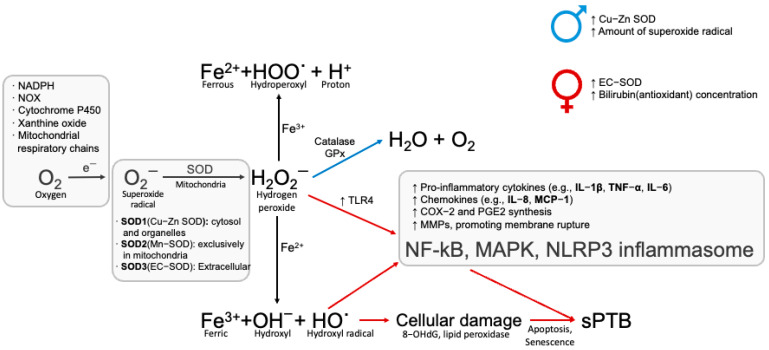
Reactive oxygen species (ROS) generation and signaling pathways. ROS are generated during cellular aerobic metabolism as a result of oxygen (O_2_) consumption. Superoxide radicals (O_2_^−^) are produced by the single electron (e^−^) reduction of O_2,_ catalyzed by enzymes such as NADPH oxidase, xanthine oxidase, and as a byproduct of mitochondrial electron transport. Superoxide dismutase (SOD) catalyzes the dismutation of O_2_^−^ into hydroxyl peroxide (H_2_O_2_). H_2_O_2_ can stimulate signaling pathways such as NF-κB, MAPK, and NLRP3 inflammasome, which are associated with inflammatory responses and may induce parturition through the expression of cyclooxygenase (COX)-2 and pro-inflammatory cytokines. H_2_O_2_ can also be converted to more ROS, such as hydroperoxyl (HOO˙) and hydroxyl radicals (HO˙) via the Fenton reaction. Antioxidant enzymes like catalase and glutathione peroxidase (GPx) degrade H_2_O_2_ into water (H_2_O), limiting oxidative stress. Excess ROS can stimulate TLR-4 signaling and cause DNA damage, such as by the oxidized derivative of deoxyguanosine, 8-oxodeoxyguanosine (8-OHdG), and lipid peroxidation [128,129,130,131].

When the balance between ROS production and antioxidant defenses is disrupted, oxidative stress can lead to a cascade of cellular damage that contributes to several pregnancy complications, including sPTB, PE, embryo resorption, intrauterine growth restriction (IUGR), and spontaneous abortion [11,127] (Figure 4).

Increased maternal blood flow within the placenta has been associated with the elevated production of ROS, resulting in lipid peroxidation, mitochondrial dysfunction, and DNA damage [130]. Among oxidative biomarkers, hydroxylated nucleotide 8-hydroxydeoxyguanine (8-OHdG) is considered an indicator of ROS-induced genotoxic stress [132]. Clinical studies have reported higher levels of 8-OHdG, malondialdehyde (MDA), and catalase activity in women with PTB compared with those with term deliveries [122,133,134,135]. These findings are accompanied by reduced antioxidant defense capacity, further confirming the role of oxidative imbalance in PTB pathophysiology [135].

Not every aspect of how ROS stimulates inflammation during pregnancy is clearly understood. ROS is known to activate the p38-mitogen-activated protein kinase (MAPK) pathway, ultimately triggering the labor process by generating uterotonic biomolecule signals (Figure 4). This pathway plays a role in the senescence of the placental fetal membrane and the pathogenesis of pPROM and PTB [122].

Despite the growing evidence regarding the contribution of oxidative stress to pregnancy complications, limited research has been undertaken to elucidate how these mechanisms may vary based on fetal sex. Understanding the sex-specific responses to oxidative stress could provide novel insights into differing pregnancy outcomes and fetal adaptation.

### 5.3. Fetal Sex Differences in Oxidative Stress Responses

The impact of oxidative stress response appears to differ by fetal sex. The mother and female fetuses showed a higher antioxidant capacity and lower oxidative stress markers than those of male fetuses. Female neonates had higher elevated antioxidant enzyme activity (catalase, GPx, and SOD) and reduced pro-inflammatory cytokines (IL-6, TNF-α), indicating a better oxidative and inflammatory balance [136]. Experimental studies in rodent models have shown that both male and ovariectomized female rats exhibit elevated levels of peroxidase and reduced mitochondrial glutathione (GSH). Interestingly, estrogen replacement restored GSH levels in ovariectomized females to those of intact females, implicating estrogen as a key mediator of antioxidant defenses [137].

It is widely accepted that the level of oxytocin in blood is higher in females compared with males [138]. The higher level of oxytocin might be the underlying factor that affects the antioxidant reaction. Furthermore, the increased total antioxidant capacity of healthy female neonates is consistent with the higher GPx activity in adult female erythrocytes compared with males [136,139]. This lower oxidative stress in females might be affected by estrogen, showing a positive relationship between estrogen and GPx [140]. Similarly, estradiol stimulates the activation of MAPK and NF-κB through the upregulation of SOD and GPx [140].

O_2_ availability and regulation are crucial in placental and fetal development, influencing the maintenance of trophoblast stemness, the regulation of proliferation and invasion, hormone synthesis, and transporter activity expression. Female fetuses tend to have higher oxygen O_2_ concentrations in the umbilical vein, which may reflect more efficient placental oxygen delivery, while a lower oxygen concentration in the umbilical artery suggests a better utilization efficiency or reduced oxygen consumption [141]. As described earlier, a high metabolic rate for the growth-priority strategy of males might generate a higher concentration of oxidative stress in males [142]. The transcriptomic analysis conducted by Lien et al. and Akram et al. supports this by showing dysregulated nutrient sensing, metabolic signaling, and catabolic processes in only the female placenta [143,144].

These differences extend to birth outcomes. Cord arterial blood in female neonates shows significantly higher levels of catecholamines in response to hypoxic stress labor, suggesting enhanced physiological adaptation mechanisms compared with males [145]. This pattern has been confirmed in animal models, where female rat pups exposed to anoxia exhibited heightened catecholamines responses, indicating sex-specific resilience under hypoxic conditions [146].

These findings collectively suggest that female fetuses may possess superior adaptive mechanisms to withstand oxidative and hypoxic stress, potentially contributing to their generally more favorable outcomes in compromised pregnancies. The higher catecholamine response in females may act as a protective mechanism to enhance survival and adaptation in utero, especially in the context of preterm labor and placental dysfunction [145].

## 6. Additional Fetal Sex-Specific Mechanisms in PTB

Glucocorticoids play a critical role in placental and fetal development, particularly in modulating stress responses through glucocorticoid receptor (GR) α-mediated signaling. Excessive glucocorticoids are associated with asthma, fetal growth, and metabolic processes [147,148]. The female fetal–placenta unit is more sensitive to cortisol via increased GRα activity through an interaction with GRα C and GRα D3 [147]. In contrast, male human and sheep placentas present significantly decreased levels of GRα and a high concentration of antagonistic isoform GRβ, which interferes with the activity of GRα and is related to glucocorticoid resistance (Figure 3) [149,150]. In the female rat placenta, the labyrinth zone exhibits a higher expression of GR (Nr3c1) [151]. In another mouse model, it was shown that the male placenta exhibited elevated levels of Nr3c1, vascular endothelial growth factor (Vegf) A, *IGF* type 1 receptor (Igf1r), and Igf2r, and slc38a1 mRNA levels during the late gestational period [151]. Conversely, female placentae demonstrated augmented levels of *Igf2* and *Slc2a1* mRNA expression, indicating that similar pathways are regulated by placental sex [151,152].

Elevated maternal cortisol levels stimulate the production of placental corticotropin-releasing hormone (CRH), creating a positive feedback loop that amplifies CRH and cortisol concentrations, ultimately contributing to increased estrogen production and the onset of PTB [152]. In addition to stimulating cortisol, CRH also enhances the placental production of estrogen. Oaks et al. found that higher cortisol levels in early and mid-pregnancy were linked to a shorter duration of pregnancy, while elevated cortisol levels at the onset of pregnancy increased the risk of PTB. The risk was three times higher in women carrying a male fetus, but not in those carrying a female fetus [153].

A heightened proportion of singleton male births in PTB has been observed, particularly during the period of 20–37 weeks of gestation. This excess ratio for males was found to be 7.2% and 2.8% in the singleton White and singleton Black groups, respectively (*p* < 0.001) [154]. Several studies have conducted genome-wide association research to identify any variants associated with PTBs or gestational age. EBF1, EEFSEC, AGTR2, ADCY5, RAP2G, and WNT4 (known to alter the binding of the estrogen receptor) loci were associated with gestational week, and the common variations in three loci (EBF1, EEFSEC, and AGTR2) were associated with PTBs in the European ancestry database [155]. However, the genome-wide association studies (GWASs) based on the Japanese population has not found significant variants [156]. The Jewish cohorts who carry a mutation of BRCA1/2 showed a significantly lower male-to-female infant ratio [157]. Despite ongoing genetic studies, the precise contribution of sexually dimorphic genetic factors to PTB remains inconclusive, highlighting the complexity of its etiology and the need for further integrative research.

## 7. Conclusions

Preterm birth is a multifactorial condition influenced by inflammation, oxidative stress, placental function, and fetal sex-specific physiological adaptation. The accumulating evidence shows that male fetuses are more vulnerable to adverse intrauterine conditions, contributing to their higher risk of PTB and poorer neonatal outcomes. In contrast, female fetuses exhibit an enhanced antioxidant capacity, stress resilience, and more efficient placental adaptations. These sexually dimorphic responses are especially relevant in the context of inflammation, placental dysfunction, and oxidative stress—three interconnected yet independently major pathological processes. Although this review does not comprehensively address all pregnancy mechanisms, it supports a growing consensus that fetal sex is one of the major key biological variables in placental development and pregnancy outcomes. Future research should prioritize fetal sex as a critical biological variable to refine prevention strategies and reduce disparities in perinatal health.

## Figures and Tables

**Table 1:** The various environmental factors related to pregnancy complications.

Environmental Factors	Exposure Window	Study (Author, Year)	Cohort (Ethnicities)	Sample Size	Trend in Fetal Sex	Significance	Key Findings/Conclusion
NO_2_	T2	Cossi et al., 2015 [28]	San Joaquin Valley of California (Hispanic)	253,704	M > F	*p* < 0.01	Exposure to NO_2_ during T2 was associated with a high risk of PTB (GW 20–27) in M infant.
PM_2.5_	Entire, T1	Park et al., 2023 [29]	Retrospective birth cohort (Korean)	1880	M > F	*p* = 0.01, *p* < 0.01	The higher risk of LBW was associated with exposure to PM_2.5_ during T1 (OR:1.05 [95% CI: 1.01–1.10]) and T2 (OR: 1.07 [95% CI; 1.03–1.12]).
Smoking	Entire (Survey)	Günther et al., 2020 [30]	Database of Schleswig-Holstein (German)	220,339	M > F	*p* < 0.001	The rate of PTB subdivided into the smoking severity. M > F for nonsmokers; M > F for: 1–7 cigarettes/day; M > F: 8–14 cigarettes/day; M > F: 15–21 cigarettes/day; M = F: ≥22 cigarettes/day.
Smoking	Entire (Survey)	Voigt et al., 2006 [31]	German birth statistics from Deutsche Perinatalerhebung (German)	888,632	M < F	*p* < 0.001	Severe smokers (>21 cigarettes/day) have a higher risk for SGA in F (3.51-fold) and in M (3.15-fold) vs. non-smoker. In mild smokers (1–5/day), the risk of SGA was 1.7275-fold in F, but was 1.7143-fold in M.
Alcohol	Not suggested	Flannigan et al., 2023 [32]	Canada	2574	FASD w/wo SFF: M = F NDF: M > F EP: M < F	*p* < 0.001	M = F: FASD diagnostic F: ↑EP anxiety, ↑depressive/mood disorders, ↑trauma. M: ↑NDF impairment, ↑ADHD, ↑conduct disorder, ↑oppositional, ↑defiant disorder. The differences were clearest in adolescents (13–17 years) and adults (≥25 years).
Heat waves	4-day (or 7-day) ^#^	Darrow et al., 2024 [33]	National Vital Statistics System at the National Center for Health Statistics Data (^##^)	55,748,869	M < F	RR (95% CI) F: 1.011 (1.001–1.020) M: 1.006 (0.997–1.015)	Subgroup analysis of RR per 1 °C increase. F: PTB and early PTB > 1 M: PTB RR > 1.
Extreme temperature	1 to 2 weeks before delivery	Yu et al., 2023 [34]	Large-scale multicenter study (Chinese)	82,221	M < F	OR (95% CI) ^###^ Fifth: 1.09 (1.04, 1.13) 10th day: 1.07 (1.04, 1.12) 10th 2D: 1.13 (1.04, 1.23)	F > M: cold spells; heat waves, ↑northern and western regions in China. Exposure to cold spells was relevant with ↑risk of PTB, especially late.

^#^ over the threshold during the exposure window, a continuous variable calculated as the 4-day (or 7-day) moving mean—the 97.5% threshold. ^##^ Hispanic or non-Hispanic ethnicity and Alaska Native, American Indian, Asian, Black, Other Pacific Islander. ^###^ 5th days, 10th days, and 10th 2D of 1 week before delivery. Abbreviations: ADHD, attention deficit hyperactivity disorder; CI, confidence interval; EP, endocrine problem; F, female; FASD, fetal alcohol spectrum disorder; GW, gestational week at; LBW, low birth weight; OR, odds ratio; *p*, *p*-value; PTB, preterm birth; SGA, small gestational age; PM_2.5_, particulate matter 2.5; RR, risk ratio; T1, 1st trimester; T2, 2nd trimester; M, male; NDF, neurodevelopmental functioning; NO_2_, nitrogen dioxide.

## Data Availability

No new data were created or analyzed in this study.

## Abbreviations

The following abbreviations are used in this manuscript:

8-OHdG	8-oxodeoxyguanosine
AG	androgen receptor
BPD	bronchopulmonary dysplasia
CCL	C-C motif chemokine ligand
COX-2	cyclooxygenase-2
DAMPs	damage-associated molecular patterns
DOHaD	developmental origins of health and disease
E	embryonic day
EVs	extracellular vesicles
Fe^2+^	ferrous iron
GWAS	genome-wide associated studies
GM-CSF	granulocyte–macrophage colony stimulating factor
GnRH	gonadotropin-releasing hormone
GPX	glutathione peroxidase
GR	glucocorticoid receptor
GRER1	G-protein-coupled estrogen receptor 1
GSH	glutathione
H_2_O_2_	hydrogen peroxide
hCG	human chorionic gonadotropin
HIF	hypoxia-inducible factor
HO	heme oxygenase
HO˙	hydroxyl radical
IFN	interferon
Ig	immunoglobulin
IGF	insulin-like growth factor
IL	interleukin
IUGR	intrauterine growth restriction
MAPK	p38-mitogen-activated protein kinase
MDA	malondialdehyde
MIAC	microbial invasion of amniotic cavity
MMP	matrix metalloproteinase-9
NF-κb	nuclear factor kappa-light-chain-enhancer of activated B cell
NK	natural killer
nRBCs	nucleated red blood cells
O_2_	superoxide radical
P4	progesterone
PAMPs	pathogen-associated molecular patterns
PAPP	pregnancy-associated plasma protein
PE	preeclampsia
PG	prostaglandin
PlGF	placental growth factor
PIVH	periventricular–intraventricular hemorrhage
pPROM	preterm premature rupture of the membrane
PRA	progesterone receptor A
PTB	preterm birth
RDS	respiratory distress syndrome
ROS	reactive oxygen species
SOD	superoxide dismutase
SpAs	spiral arteries
sPTB	spontaneous preterm birth
SRY	sex-determining region
T1	trimester 1
T2	trimester 2
T3	trimester 3
TLR	toll-like receptor
TNF	tumor necrosis factor
Vegf	vascular endothelial growth factor
XCI	X chromosome inhibition
